# Identification and characterization of microRNAs in *Clonorchis sinensis *of human health significance

**DOI:** 10.1186/1471-2164-11-521

**Published:** 2010-09-28

**Authors:** Min-Jun Xu, Quan Liu, Alasdair J Nisbet, Xian-Quan Cai, Chao Yan, Rui-Qing Lin, Zi-Guo Yuan, Hui-Qun Song, Xian-Hui He, Xing-Quan Zhu

**Affiliations:** 1 Department of Parasitology, College of Veterinary Medicine, South China Agricultural University, Guangzhou, Guangdong Province 510642, PR China; 2 State Key Laboratory of Veterinary Etiological Biology, Key Laboratory of Veterinary Parasitology of Gansu Province, Lanzhou Veterinary Research Institute, CAAS, Lanzhou, Gansu Province 730046, PR China; 3 Laboratory of Parasitology, Veterinary Institute, AMMS, 1068 Qinglong Road, Changchun 130062, PR China; 4 Parasitology Division, Moredun Research Institute, Pentlands Science Park, Midlothian EH26 0PZ, UK; 5 Zhongshan Entry-Exit Inspection and Quarantine Bureau, Zhongshan, Guangdong Province 528403, PR China; 6 College of Animal Science and Technology, Yunnan Agricultural University, Kunming, Yunnan Province 650201, PR China

## Abstract

**Background:**

*Clonorchis sinensis *is a zoonotic parasite causing clonorchiasis-associated human disease such as biliary calculi, cholecystitis, liver cirrhosis, and it is currently classified as carcinogenic to humans for cholangiocarcinoma. MicroRNAs (miRNAs) are non-coding, regulating small RNA molecules which are essential for the complex life cycles of parasites and are involved in parasitic infections. To identify and characterize miRNAs expressed in adult *C. sinensis *residing chronically in the biliary tract, we developed an integrative approach combining deep sequencing and bioinformatic predictions with stem-loop real-time PCR analysis.

**Results:**

Here we report the use of this approach to identify and clone 6 new and 62,512 conserved *C. sinensis *miRNAs which belonged to 284 families. There was strong bias on families, family members and sequence nucleotides in *C. sinensis*. Uracil was the dominant nucleotide, particularly at positions 1, 14 and 22, which were located approximately at the beginning, middle and end of conserved miRNAs. There was no significant "seed region" at the first and ninth positions which were commonly found in human, animals and plants. Categorization of conserved miRNAs indicated that miRNAs of *C. sinensis *were still innovated and concentrated along three branches of the phylogenetic tree leading to bilaterians, insects and coelomates. There were two miRNA strategies in *C. sinensis *for its parasitic life: keeping a large category of miRNA families of different animals and keeping stringent conserved seed regions with high active innovation in other places of miRNAs mainly in the middle and the end, which were perfect for the parasite to perform its complex life style and for host changes.

**Conclusions:**

The present study represented the first large scale characterization of *C. sinensis *miRNAs, which have implications for understanding the complex biology of this zoonotic parasite, as well as miRNA studies of other related species such as *Opisthorchis viverrini *and *Opisthorchis felineus *of human and animal health significance.

## Background

Fish-borne clonorchiasis, caused by the oriental liver fluke *Clonorchis sinensis*, is endemic in many Asian countries and over 35 million people globally are infected with *C. sinensis*, including an estimated 15 million in China [1]. The parasite has major socioeconomic impacts in other parts of Asia as well. In Korea, infection in humans is one of the most prevalent [2]. While in Vietnam, the prevalence reaches 79% in the Haiphong and Hanoi area [3]. This infection is also becoming increasingly common in non-endemic regions and in developed countries due to growing international markets, improved transportation systems, and demographic changes such as population movements [2,3]. Epidemiological data suggested that clonorchiasis has an increasing human-health impact resulted from the greater consumption of raw, frozen, dried, or pickled freshwater fish imported from endemic areas [1,4].

Adult *C. sinensis *flukes reside chronically in the biliary tract and cause periductal inflammation, fibrosis, pyogenic cholangitis, biliary calculi, cholecystitis, liver cirrhosis and pancreatitis [2,5]. Like *Opistorchis viverrini*, *C. sinensis *is classified as carcinogenic to humans by the International Agency for Research on Cancer for cholangiocarcinoma in 2009 [6]. The disease arises from metaplastic changes of biliary epithelial cells and usually occurs in the secondary intrahepatic bile duct, where the fluke is preferentially situated. However, the exact mechanisms of the carcinogenesis are not clearly elucidated [3,7].

The prevention and control strategies for this parasite include fecal examination and treatment of individual patients with praziquantel. The World Health Organization has also recommended mass chemotherapy in humans in endemic areas as the most practical and immediately effective control strategy [2]. Other efforts to control the parasite include interrupting transmission at the intermediate host level. However, there has been little effect on the impact of snail populations (the first intermediate host of the parasite) or on the practice of eating raw fish [1].

MicroRNAs (miRNAs) are 18-22 nucleotide, non-coding, small RNA molecules found in diverse organisms from viruses [8], plants [9], flies [10] to mammals [11,12], which regulate gene expression at the post-transcriptional level. They are essential for the complex life cycle of pathogenic parasites for their ability to respond to environmental and developmental signals and are now considered as a key mechanism of gene regulation [13]. The discovery of miRNA function sheds new light on the control of these parasites. However, there was no miRNAs being identified experimentally in *C. sinensis*.

In light of the probability that miRNA species are involved in gene regulation in *C. sinensis*, here we investigated the expression profile of miRNAs and detected potential novel miRNAs in *C. sinensis *adults. Due to the similarity in morphology, life cycle and modes of transmission among members of the Opisthorchiidae [3], miRNA profile research in *C. sinensis *will shed light on the miRNA studies of other species such as *O. felineus *and *O. viverrini*.

## Results

### Profile characteristics of short RNAs from *C. sinensis*

**Figure 1:**
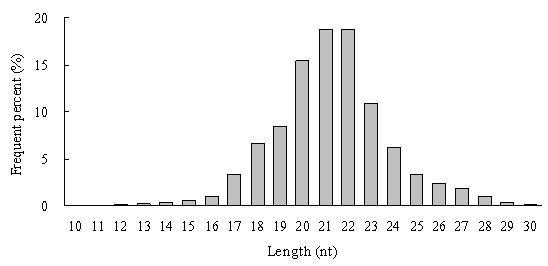
**Length distribution of small RNAs from *Clonorchis sinensis *identified and analyzed by deep sequencing**. Analysis of 12,138,350 high quality reads after filtering low quality tags, 5' and 3' adaptor and contamination formed by adaptor-adaptor ligation.

Deep sequencing yielded 14.8 million reads (Additional file 1 for flowchart), and the raw sequencing data was deposited in GEO of NCBI http://www.ncbi.nlm.nih.gov/geo/ with accession number GSE22244. After 5' and 3' adaptors, contamination formed by adaptor-adaptor ligation and low quality tags were removed, a total of 12.14 million reads with high quality were obtained. Length distribution analysis showed that most reads were distributed among 20-23 nt. The highest percentage was 18.73% with reads of 21 nt long, followed by 18.68% of 22 nt reads (Figure 1). In the next analysis step, reads smaller than 18 nt (6.05%) were removed, and a total of 11.19 million clean reads remained with 2.74 million (24.44%) unique sequences. Among the 11.19 million clean reads, a total of 2.18 million (19.46%) were perfectly mapped to the *Schistosoma japonicum *genome, including 30,558 (1.12%) unique sequences; and 16.13% of these have only one location on the genome.

Among the 11.19 million reads, 1,222,173 (10.92%) were ncRNAs, including rRNA, tRNA, snRNA and snoRNA. Repeat-associated small RNAs (1,909; 0.02%) coming from high-repeat regions of genome or transposon-regions were found to belong to the two types of repeat: LINE/RTE:0 and LINE/RTE:1. The percentage of known miRNAs was 18.71% with 2,093,879 reads which includes 62,512 unique sequences (Additional file 2). Except for the miRNA, rRNA and repeats mentioned above, 7,875,451 (70.36%) sequences (2,610,289 unique reads) had no match and were marked as un-annotated (Table 1).

**Table 1:** Summary of reads that match various RNAs

Locus class	Unique reads	Total reads
rRNAetc	61946 (2.26%)	1222173 (10.92%)
Repeat	732 (0.03%)	1909 (0.02%)
Known miRNAs	62512 (2.29%)	2093879 (18.71%)
other small RNAs	2610289 (95.42%)	7875451 (70.36%)
Total	2735479 (100%)	11193412 (100%)

### Identification of miRNA* and 6 novel miRNAs

**Figure 2:**
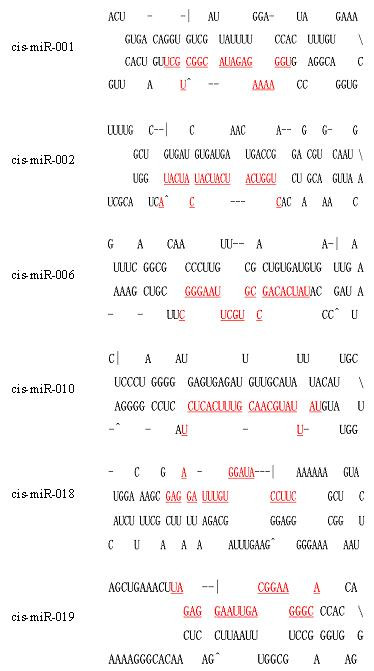
**The predicted stem-loop structures for the six novel miRNA precursors in *Clonorchis sinensis***. The mature miRNA sequences are shown in red and underlined. The actual size of each putative precursor was not identified experimentally and may be slightly shorter or longer than represented. There are two possible stem-loop structures for *cis-miR-006 *and three predicted stem-loop structures for *cis-miR-019*, the structures of these two precursors shown in this figure are the ones recommended by the software of Mfold.

A total of 17,535 un-annotated unique reads (out of 30,558 unique ones) that can match elements of the *S. japonicum *genome were marked as potential novel miRNA candidates. The secondary structure of the inverted repeat predicted by Mfold and evaluated by MirCheck showed that 6 conserved reads were found (Additional files 3, 4). Many miRNAs were found in multiple locations of the *S. japonicum *genome, for example, a novel miRNA named *cis-miR-001*, corresponds to 14 different locations on different chains of the genome (Additional file 5). Although we found the homologs of known miRNA* of other organisms, we did not obtain any miRNA* sequences of this 6 novel miRNAs in the *C. sinensis *data set. The sequences and locations of the 6 predicted novel reads are shown in Table 2 and the predicted stem-loop structures for their miRNA precursors are shown in Figure 2.

**Table 2:** Sequences of the six novel miRNAs identified in Clonorchis sinensis and their location within the published Schistosoma japonicum genome

miRNA	Sequence (5'-3')	Size	Loci^a^	Count^b^	ΔG^c^	Express level^d^
cis-mir-001	UGGAAAAGAGAUACGGCUGCU	21	14	12	-23.1	0.30 ± 0.07
cis-mir-002	CUGGUCAUCAUCAUCAUCAUA	21	1	5	-23.0	0.10 ± 0.02
cis-mir-006	UAUCACAGCCGUGCUUAAGGGC	22	1	108	-28.4	0.001 ± 0.00
cis-mir-010	UAUUAUGCAACGUUUCACUCU	21	1	8	-37.9	0.02 ± 0.01
cis-mir-018	GAGAGAUUUGUGGAUACCUU	20	2	13	-21.9	0.0005 ± 0.00
cis-mir-019	UAGAGGAAUUGACGGAAGGGCA	22	1	5	-19.9	67.32 ± 12.87

^a ^Location number of the miRNA sequence with the published genome sequence of *S. japonicum *of LSBI, Shanghai.

^b ^The number of each miRNA appeared in the clean reads.

^c ^ΔG means the energy of pre-miRNA hairpin, kcal/mol.

^d ^The expression levels of the six novel miRNA relative to actin gene, and the data represent the means and standard deviation (SD) for triplicate reactions independently.

Most miRNA* homologs had only one copy in the *C. sinensis *dataset and the most abundant unique reads was homologs of gga-miR-1627* with only 238 counts (Additional file 6). Although there were abundant miRNA* sequences in *Caenorhabditis elegans *and other organisms in Sanger miRBase [14] (Release 13), all these miRNA* homologs belonged to different kinds of organisms including vertebrates, insects, virus and some coelomates, such as *Amphimedon queenslandica*, *Schmidtea mediterranea*, *Locusta migratoria*, *Xenopus tropicalis*, *Mus musculus *and *Gallus gallus*, with the exception of parasites and nematodes including S*chistosoma mansoni*, *S. japonicum*, *C. elegans*, *C. briggsae *and all kinds of mosquitoes including *Anopheles gambiae*, *Aedes aegypti *and *Culex quinquefasciatus*, which were deposited in the database (Additional file 6). It was reported that though the miRNA: miRNA* duplex were complementary, their base-pairing was imperfect and miRNA* was less stable than the mature miRNA [15]. The phenomenon above might indicate a fast degradation mature mechanism of miRNA* in parasites, at least in *C. sinensis*.

### The phylogenetic evolution of miRNAs

**Figure 3:**
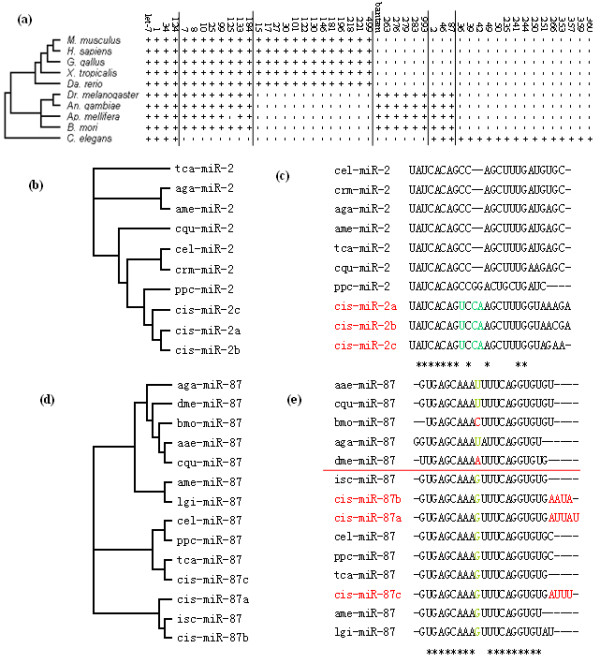
**Phylogenetic evolution of 50 conserved miRNA families in *Clonorchis sinensis***. (a) Phylogenetic distribution of 50 conserved miRNA families of *C. sinensis*. A plus (+) symbol indicates this miRNA family exists in the species named on the left. (b and c) Phylogenetic analysis of *miR-2 *family shows middle evolution approach of miRNA in *C. sinensis*. (d and e) Phylogenetic analysis of *miR-87 *family shows another evolution approach of miRNA in *C. sinensis *which is conserved in middle and changed in the tail.

Some families included many members and showed distribution bias in the *C. sinensis *dataset. Totally, 284 conserved miRNAs families were found in *C. sinensis *out of 2,093,879 reads (with 62,512 unique sequences). These conserved families were presented in large category of vertebrates, insects and nematodes, and can be sorted into 6 groups based on their phylogenetic distribution. Fifty conserved miRNA families are showed in Figure 3a as examples. Four families were found to present among vertebrates, insects and nematodes, including *let-7*, *miR-1*, *miR-34 *and *miR-124*; Some families were present in vertebrates and insects, but no nematodes; Some were restricted respectively to vertebrates, insects, and invertebrates (insects and nematodes); while some were nematode-specific. This phenomenon showed that *C. sinensis *miRNAs were distributed widely, some of which specifically belonged to other animals and there might be a redundant miRNA expression in *C. siensis*. It was reported that there were 50 conserved families and 185 potential locust-specific miRNA families in locust, and these families can only be divided into 4 groups [15]. It was also reported that 16 miRNAs belonged to 13 miRNA families in *S. japonicum *[16], but no detailed information about miRNA families' distribution was reported. Considering the complex parasitic life of *C. sinensis *and the key regulation function of miRNAs, possessing miRNAs of different kinds of animals would be a perfect strategy for its parasitic life, which could help them to adapt and modify the host and parasitic environments quickly and conveniently. On the other hand, for a large number and kinds of miRNA expressed, nucleotides of miRNA* need to be reused quickly, and this might be the reason why there were so little miRNA* sequences found in this kind of parasite. It is known that *C. sinensis *is parasitic in animal hosts such as snails, cats, dogs, fishes, mice and human beings. However, due to the widely distribution of its miRNA families, we believe that this parasite can easily enlarge its host range when the parasitic situation is convenient.

Categorization of conserved miRNAs indicated that miRNAs of *C. sinensis *were still in the process of evolution. Some of the miRNAs belonged to ancient families, such as *miR-1 *and *miR-34*; while some others appeared to be much younger, such as *C. sinensis *specific miRNAs. The innovation was concentrated along three branches of the phylogenetic tree leading to bilaterians, insects and coelomates. Age differences indicated that there was an ongoing process of miRNA evolution and the birth and death of a kind of miRNA family was a common phenomenon [15].

There might be two innovation approaches of miRNA in *C. sinensis*, including evolution in the middle and tail. We randomly elected 4 conserved families, *miR-2*, *miR-7*, *miR-133 *and *miR-87*, and analyzed all of the members of each in miRBase. *miR-7 *and *miR-133 *belonged to vertebrates, insects and nematodes, while *miR-2 *and *miR-87 *were present in invertebrates only (Figure 3a). If a miRNA family of *C. sinensis *had more than one of its members, we chose the most abundant 2 or 3 ones, for they were the main part of the family. Phylogenetic analysis of *miR-2 *family showed that the *cis-miR-2 *can be easily found for its substitutions and insertions in the middle of miRNA, just behind the "seed region" (Figure 3c); In contrast, the *cis-miR-87 *was conserved in the middle, and mosquitoes (above red line) and some other insects including *B. mori *and *D. melanogaster *innovated at the position. However, it can be found that *cis-miR-87 *evolved from the tail (Figure 3e). Different members of the *cis-miR *family showed different innovation rate indicated by phylogenetic trees (Figure 3b, d). The same phenomenon can also be found in families of *miR-7 *and *miR-133 *(Additional file 7). It was found that the seed regions of all the species were conserved, while one nucleotide right behind the seed region was changed from U to G and another one was deleted in *cis-miR-7 *of *miR-7 *family. In *miR-133*, one nucleotide was changed from U to C in the middle which was the same as *sja-miR-133 *(*S. japonicum*), and another one was changed from U to A which was the same as *bma-miR-133 *(*B. malayi*). We supposed that there might be a kind of site-directed evolution and mutagenesis for parasitic life in parasites, however, more miRNA information of other kinds of parasites are needed, which are absent in public databases at present.

### Strong family member bias of miRNAs in *C. sinensis*

Almost one third of the reads belonged to *miR-71 *family with a total percentage of 33.24%, which included *miR-71 *(26.79%), *miR-71a *(0.01%), *miR-71b *(0.03%) and *miR-71c *(6.41%). It was followed by the family *miR-2 *with most of the reads focusing on *miR-2b*. This bias phenomenon has also been found in other families, such as *miR-277 *and *miR-1*. Except for *miR-1 *which was common in different kinds of animals, miRNAs including *miR-71*, *miR-2 *and *miR-277 *all belonged to invertebrates (insects and nematodes).

Some families included more members than others. *let-7 *was the second miRNA found in nematode *C. elegans *in 2000 [17] and it was found that the *let-7 *family was the largest one among the conserved miRNAs of *C. sinensis*, including 15 members from *let-7a *to *let-7j *and star sequences such as let-7b* and let-7g*. The *miR-1422 *family was the second largest family represented in the current dataset, which included 13 members. The reads in these families showed strong bias. For example, 95.12% of the reads focused on *let-7 *in the *let-7 *family.

### Expression predominance of some miRNAs in *C. sinensis*

Some kinds of miRNAs were expressed with high predominance. *miR-71 *had the most abundant reads accounting for 26.79% (597,871) of the total reads. It was followed by *miR-277b *with a percentage of 7.97% (98,845 reads). The third most abundant miRNA is *miR-71c *with a percentage of 6.41% (Additional file 8). The miRNA named *lin-4*, which was firstly found in the nematode *C. elegans *in 1993 [18], was also found in *C. sinensis *(with only 5 reads). Some other miRNAs were not found in *C. sinensis*, including *miR-40*, *miR-46*, *miR-79*, and *miR-103*.

There were 756 kinds of miRNA with copy numbers (reads) fewer than 1,000, and 45 kinds of them with only 1 copy. Seventy-eight kinds of miRNAs had reads of between 1,000 and 10, 000, while there were only 30 kinds of miRNA with copy numbers between 10,000 and 100,000. When the copy numbers were higher than 100,000, only 4 kinds of miRNA were represented (*miR-71*, *miR-277b*, *miR-71c *and *miR-215*). This expression predominance phenomenon was also found in the 6 novel miRNAs. For example, there were 108 copies of *cis-miR-006*, while only 5 copies of *cis-miR-002 *and *cis-miR-019 *were found, respectively (Table 2).

### Nucleotides bias of miRNAs in *C. sinensis*

**Figure 4:**
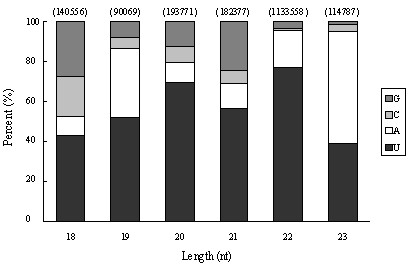
**Analysis of first nucleotide bias of miRNAs in *Clonorchis sinensis***.

The first nucleotide bias analysis of the 2,093,879 reads revealed that nucleotide uracil (U) was the most frequently used first nucleotide in miRNAs of *C. sinensis *(70.07% incidence), while G or C was seldom used as the first nucleotide with only 10.31% and 3.32%, respectively (Figure 4). (A+U) was found most abundantly with a percentage of 86.37% on average, reaching a percentage of 95.75% and 95.23% at the 22 and 23 nt positions, respectively (Figure 4).

Nucleotide bias analysis at each position showed that A and U mainly appeared at the beginnings and the ends of reads, while C and G occupied a very high percentage at the second position and the positions of 6-9th, which belonged to the "seed region" in other species [19,20]. Particularly at the beginning of seed region, the G+C content reached as high as 80.06% (Additional file 9). For a broad range of miRNA families which were presented in *C. sinensis *(Figure 3a), the seed regions of them might tend to be conserved. This speculation can be proven by the phylogenetic analysis of the two families of *miR-2 *and *miR-87 *(Figure 3). Although there were innovations to the families of *miR-2 *and *miR-87 *in *C. sinensis*, nucleotide change only happened in the middle and the end. Considering the complex life history of the parasite, keeping steady seed region and flexible in other places of miRNA might be another strategy for the parasite to adapt to different parasitic situations in different tissues of different hosts.

The analysis showed that U had a high frequency in the 1st, 14th and 22nd positions with percentages of 68.01%, 61.38% and 66.43% respectively, while it seldom appeared at the 2nd and 24th positions with percentages of 3.80% and 2.44%. (A+U) was distributed mainly in the front of reads, at the first and 3-5th nucleotides (86.76%, 80.67%, 69.92%, and 79.84% respectively) but less at the second position (19.94%). C had the lowest percentage (1.73%) at 15th position. G showed high percentage incidence at the positions of almost every 3 nucleotides, such as 6th, 12th and 18th with 69.54%, 50.77% and 58.61%, respectively, and it seldom appeared at the end positions, such as the 22nd position (0.93%). The highest percentage of (G+C) was located at the second position with 80.06%, and it also predominated at the positions of 6-9th and 16-18th. The phenomenon that most A and U were distributed in the front of reads with G and C being focused on the 6-18 positions might be concerned with the mechanisms of miRNA action, such as binding with the targets for gene regulation.

### Quantification of *C. sinensis *miRNA expression

Using the modified stem-loop RT-PCR, the relative expression levels of the six novel miRNAs relative to the actin gene were calculated (Additional file 10, Table 2, expression level). Among them, *cis-miR-019 *showed a high expression level while that of *cis-miR-018 *and *cis-miR-006 *were very low.

## Discussion

miRNAs are now considered as key regulators of gene expression at the post-transcriptional level and perform a variety of significant functions within cells such as regulation of growth, metabolism, development and cell differentiation [21-23]. Due to the complex life cycle of parasites with several developmental stages in vertebrate and invertebrate hosts, it is particularly important to elucidate the roles of miRNAs in the growth and development of parasites and their abilities to regulate infection of mammalian hosts. miRNAs are involved not only in the normal functioning of eukaryotic cells, but also associated with dysregulation during disease. miRNAs can be used as potential new tools for disease diagnostics and gene therapy [24] and a manually curated database (miR2Disease) is publicly available aiming at documenting known relationships between miRNA dysregulation and human disease [25].

miRNAs in *C. sinensis *of zoonotic significance were identified and characterized by deep sequencing in the present study. We found that the percentage of reads matching the *S. japonicum *genome was very low (19.46%) and only 30,558 unique reads out of 2,735,479 were perfectly matched, whereas this figure can be as high as 70.5% or higher in some other species [26]. The most likely reason for this phenomenon might be that the reference genome used for matching analysis was the *S. japonicum *genome, rather than *C. sinensis*, for the *C. sinensis *genome (or other species of Opisthorchiidae) was not available at present. Although *C. sinensis *and *S. japonicum *belong to a common phylogenetic group (Trematoda), there are some significant differences between them, and some miRNAs appeared to be species-specific [27].

The distinguishing characteristic of miRNAs from other endogenous small RNAs is that miRNA precursors have the inverted repeat sequence that can form hairpin structures [28]. Six novel miRNAs were predicted from 17,535 un-annotated genome-matched unique reads. We cannot exclude the possibility that there might be more miRNA types in *C. sinensis *adults.

Amongst the conserved miRNAs of *C. sinensis*, bias was found within both kinds of families and family members. The *miR-71 *and its families possessed the highest proportion of total reads, and the *miR-71 *family was conserved in *S. mansoni*, *S. japonicum*, *Ixodes scapularis*, and *Anopheles gambiae *being indexed by Sanger miRBase, which indicated that *miR-71 *is essential for the life of this flatworm. Like *miR-71*, some miRNAs had particularly high copies in *C. sinensis*, including *miR-277b*, *miR-71c *and *miR-215*. Considering the living environment of the adult parasites in the biliary tract of the hosts, as well as the normal function of miRNAs, we inferred that these families might be mainly involved in metabolism of the worm.

Nucleotides A and U were distributed mainly in the front of reads including the first and the 3-5th nucleotides with the exception of the second position (80.06% of G+C). U was the dominant nucleotide in mature miRNAs, especially in the first nucleotide position, and it showed a high frequency in the 1st, 14th and 22nd position, almost at the beginning, the middle, and the end of reads. It was reported by Zhang et al. (2009) that the 1st, 9th and the terminal positions were enriched with U and the 1st and 9th positions were the limits of the "seed region" of a miRNA, which was responsible for targeting mRNAs for gene regulation [20]. We showed a similar result in *C. sinensis *miRNAs at the first and the end positions, but there was only 20.13% on average at the ninth position, while it was 50.66% and 61.38% respectively at the 11th and the 14th positions. At the positions of 6-9th, G or C occupied a high percentage. Because the previously analyzed miRNAs were mainly derived from vertebrates including human, rat, mouse and pig, and only a few parasites were involved, we cannot exclude the possibility of species- or even order-specific differences and there might be a different or shifted "seed region" for miRNAs in *C. sinensis *compared with other species [20].

## Conclusions

The present study represented the first large scale characterization of *C. sinensis *miRNAs, which will help us understand the complex life cycle of this zoonotic parasite, which in turn may have implications for the development of novel approaches for the effective control of this parasite. These results will also assist the miRNA studies of other related species such as *Opisthorchis felineus *and *O. viverrini *of human and animal health significance.

## Methods

### Parasites

Adults of *C. sinensis *were collected from the bile ducts of 8 wild cats obtained in the suburbs of Guangzhou, China, with 20 worms in average from each cat. The worms were immediately transferred to sterile physiological saline (37°C) in a sterile beaker, followed by washing five times with saline on a rotary shaker to remove contamination from the hosts. Thereafter, the flukes were transferred to Dulbecco's modification of Eagle's medium (DMEM) and incubated at 37°C with 10% CO_2 _for 3 h, allowing the flukes to regurgitate the gut contents until the flukes appeared pale and uniform. The flukes were then transferred to RNase-free 1.5 ml screw-top cryotube containing RNAlater (Sigma) and kept at 4°C for overnight, followed by storage at -70°C.

The maintenance and care of animals used in this study were handled in strict accordance with good animal practice as defined by the relevant national and/or local animal welfare bodies, and all animal work was approved by the appropriate committee.

### RNA preparation

Total RNA from flukes was prepared using Trizol reagent (Invitrogen) according to the manufacturer's protocol with some modifications. Briefly, at the step of precipitation, 100% and 50% isopropanol were used to gain a clear mix solution, followed by incubation for 1 h at -70°C instead of 5 min at room temperature to enhance the precipitation of low-molecular-weight (LMW) RNAs. The purity and integrity of total RNA were examined by standard agarose gel electrophoresis, and the concentration was determined using a BioPhotometer (Eppendorf). The purified total RNA was stored at -70°C until use.

### Small RNA isolation and high-throughput sequencing

The small RNA isolation was performed as described previously [29]. RNA fragments of 20-30 bases long were isolated and purified from 10 μg total RNA using a Novex 15% TBE-Urea gel. The 5' and 3' adaptors (Illumina) were added to the ends of fragments. Reverse transcription PCR (RT-PCR) was performed using a RT-PCR kit (Invitrogen). The fragments were purified using a 6% TBE PAGE gel and used for high-throughput sequencing with a Solexa sequencer at Huada Genomics Institute Co. Ltd, China. All the gels and kit for small RNA purification and amplification were bought from Invitrogen Co. Ltd.

### Computational analysis

After masking of adaptor sequences and removal of redundancy and reads smaller than 18 nt as well, the cleans reads were screened against GenBank and Rfam database (version 9.0) http://www.sanger.ac.uk/software/Rfam/mirna to remove non-coding RNA, such as rRNA, tRNA, snRNA, snoRNA, and other ncRNA. The sequences of candidate precursors were analyzed using RepeatMasker http://www.repeatmasker.org to eliminate the repetitive sequences and then the left reads were searched against the Sanger miRBase (version 13.0) to identify the conserved miRNAs. Based on the nomenclature of miRNAs, reads showing high similarity to known miRNAs of other organisms (mismatches ≤ 2) were classified into the same miRNA family [15,30]. The family distribution of conserved miRNA and the nucleotide bias were gathered statistically to analysis the expression and coding characters of miRNAs. Reads that cannot match any database above were marked as unannotation. Cleans reads were then mapped onto the genome of *Schistosoma japonicum *http://lifecenter.sgst.cn/schistosoma/cn/genomeProject.do using the program of Short Oligo nucleotide Analysis Package (SOAP) [31]. The perfectly matched unannotation reads were predicted with Mfold http://www.bioinfo.rpi.edu/applications/mfold and then evaluated by MirCheck. Thereafter, the precursors (hairpin) of miRNAs were inspected manually in order to remove false predictions. Predicted precursors that miRNAs and miRNA* can be found in its both arms were deemed as high probability and stem-loop hairpins were considered typical when the mature miRNAs present in one arm instead of loop of hairpin precursors and with free energy hybridization lower than -18 kcal/mol.

### Analysis of novel miRNA expression

Stem-loop real-time reverse transcription polymerase chain reaction (RT-PCR) with SYBR Green was used for the analysis of novel miRNA expression in *C. sinensis *adults. The stem-loop primers were used to quantify the miRNA expression because it can provide more specificity and sensitivity than linear primers [32]. All of the primers were synthesized by Shenggong Co, Ltd., China.

Real-time quantitative PCR was performed using an ABI PRISM^® ^7300 Sequence Detection System and SYBR Green PCR Master Mix (TOYOBO) in a 20 μl reaction. All reactions were carried out in triplicate. The PCR mix included 5 μl cDNA for each miRNA (in 1:20 dilution), 5 μM forward and reverse primers, respectively, 10 μl 2× SYBR Green PCR Master Mix. The *C. sinensis *actin gene (EU109284) was used as the endogenous control. The primer pairs were as follows: forward 5'-ATGGGTGATGAGGACGTTGCAGCT-3' and reverse 5'-CATGATCGAGTTGTA CG TCGTCTC-3' [33]. The cycle conditions were as follows: 95°C 10 min, followed by 40 cycles of 95°C for 15 s, 65°C for 30 s, and 72°C for 30 s. The threshold cycle (Ct) was defined as the cycle number at which the fluorescence intensity passed a predetermined threshold. The quantification of each miRNA relative to actin gene was calculated using the equation: N = 2^-ΔCt^, ΔCt = Ct_miRNA_-Ct_acin _[34,35].

## Authors' contributions

XQZ and MJX conceived and designed the experiments. MJX, XQC, CY and XHH performed the experiments. MJX, QL, AJN, RQL and ZGY analyzed the data. HQS and XHH contributed reagents/materials. MJX, QL, AJN and XQZ wrote the manuscript. All authors read and approved the final manuscript.

## Authors' information

^1^Department of Parasitology, College of Veterinary Medicine, South China Agricultural University, Guangzhou, Guangdong Province 510642, PR China. ^2^State Key Laboratory of Veterinary Etiological Biology, Key Laboratory of Veterinary Parasitology of Gansu Province, Lanzhou Veterinary Research Institute, CAAS, Lanzhou, Gansu Province 730046, PR China. ^3^Laboratory of Parasitology, Veterinary Institute, AMMS, 1068 Qinglong Road, Changchun 130062, PR China. ^4^Parasitology Division, Moredun Research Institute, Pentlands Science Park, Midlothian EH26 0PZ, Scotland. ^5^Zhongshan Entry-Exit Inspection and Quarantine Bureau, Zhongshan, Guangdong Province 528403, PR China. ^6^College of Animal Science and Technology, Yunnan Agricultural University, Kunming, Yunnan Province 650201, PR China.

## Supplementary Material

Additional file 1 **Analyzing flowchart of *Clonorchis sinensis *miRNAs**. Click here for file

Additional file 2 **Known miRNAs of *Clonorchis sinensis***. Click here for file

Additional file 3 **Six predicted novel miRNAs in *Clonorchis sinensis***. Click here for file

Additional file 4 **Detailed information of the six novel miRNAs in *Clonorchis sinensis***. Click here for file

Additional file 5 **Precusors of *cis-miR-001 *in *Clonorchis sinensis***. Click here for file

Additional file 6 **Known miRNA star homologs in *Clonorchis sinensis***. Click here for file

Additional file 7 **Phylogenetic analysis of the *miR-7 *and *miR-133 *families in *Clonorchis sinensis***. Click here for file

Additional file 8 **The miRNA distribution in *Clonorchis sinensis***. Click here for file

Additional file 9 **The nucleotide bias percentage at each position in miRNAs of *Clonorchis sinensis***. Click here for file

Additional file 10 **The amplification and melting curves of the six novel miRNAs of *Clonorchis sinensis *by Real-Time quantitative PCR**. Click here for file
