# Supplementary material for: Identification and characterization of microRNAs in Clonorchis sinensis of human health significance
Source: BMC Genomics. 2010 Sep 28;11:521. doi: 10.1186/1471-2164-11-521 (PMC3224684; doi:10.1186/1471-2164-11-521)
Supplement: Additional file 1 — Analyzing flowchart of Clonorchis sinensis miRNAs. [file 1471-2164-11-521-S1.DOC]

**Additional file 1：Analyzing flowchart of *Clonorchis sinensis* miRNAs.**
