# Supplementary material for: Identification and characterization of microRNAs in Clonorchis sinensis of human health significance
Source: BMC Genomics. 2010 Sep 28;11:521. doi: 10.1186/1471-2164-11-521 (PMC3224684; doi:10.1186/1471-2164-11-521)
Supplement: Additional file 8 — The miRNA distribution in Clonorchis sinensis. [file 1471-2164-11-521-S8.DOC]

**Additional file 8:** **The miRNA distribution in *C*. *sinensis*. The first 20 miRNAs with maximum numbers of reads are showed.**

**Known miRNAs that have star sequences**

a) miR-2020 is only star sequences in the C. sinensis miRNA dataset.
