# Supplementary material for: Identification and characterization of microRNAs in Clonorchis sinensis of human health significance
Source: BMC Genomics. 2010 Sep 28;11:521. doi: 10.1186/1471-2164-11-521 (PMC3224684; doi:10.1186/1471-2164-11-521)
Supplement: Additional file 9 — The nucleotide bias percentage at each position in miRNAs of Clonorchis sinensis. [file 1471-2164-11-521-S9.DOC]

**Additional file 9: The nucleotide bias percentage at each position in miRNAs of *C*. *sinensis*.**
