# Supplementary material for: Identification and characterization of microRNAs in Clonorchis sinensis of human health significance
Source: BMC Genomics. 2010 Sep 28;11:521. doi: 10.1186/1471-2164-11-521 (PMC3224684; doi:10.1186/1471-2164-11-521)
Supplement: Additional file 10 — The amplification and melting curves of the six novel miRNAs of Clonorchis sinensis by Real-Time quantitative PCR. [file 1471-2164-11-521-S10.DOC]

**Additional file 10: The amplification and melting curves of the six novel miRNAs of *Clonorchis sinensis* by Real-time quantitative PCR.**
