# Supplementary material for: Identification and characterization of microRNAs in Clonorchis sinensis of human health significance
Source: BMC Genomics. 2010 Sep 28;11:521. doi: 10.1186/1471-2164-11-521 (PMC3224684; doi:10.1186/1471-2164-11-521)
Supplement: Supplementary file 10 [file 1471-2164-11-521-S7.DOC]

```
ppy-miR-7     --UGGAAGACUAGUGAUUUUGUU------
sla-miR-7     --UGGAAGACUAGUGAUUUUGUU------
ppa-miR-7     --UGGAAGACUAGUGAUUUUGUU------
lla-miR-7     --UGGAAGACUAGUGAUUUUGUU------
mme-miR-7     --UGGAAGACUAGUGAUUUUGUU------
ggo-miR-7     --UGGAAGACUAGUGAUUUUGUU------
fru-miR-7     --UGGAAGACUAGUGAUUUUGUU------
xtr-miR-7     --UGGAAGACUAGUGAUUUUGUUG-----
odi-miR-7     --UGGAAGACUAGUGAUUUUGUUG-----
mdo-miR-7     --UGGAAGACUAGUGAUUUUGUUG-----
gga-miR-7     --UGGAAGACUAGUGAUUUUGUUG-----
cin-miR-7     --UGGAAGACUAGUGAUUUUGUUG-----
cfa-miR-7     --UGGAAGACUAGUGAUUUUGUUGU----
tca-miR-7     --UGGAAGACUAGUGAUUUUGUUGU----
tni-miR-7     --UGGAAGACUAGUGAUUUUGUUGU----
tgu-miR-7     --UGGAAGACUAGUGAUUUUGUUGU----
spu-miR-7     --UGGAAGACUAGUGAUUUUGUUGU----
sko-miR-7     --UGGAAGACUAGUGAUUUUGUUGU----
oan-miR-7     --UGGAAGACUAGUGAUUUUGUUGU----
nvi-miR-7     --UGGAAGACUAGUGAUUUUGUUGU----
isc-miR-7     --UGGAAGACUAGUGAUUUUGUUGU----
hsa-miR-7     --UGGAAGACUAGUGAUUUUGUUGU----
eca-miR-7     --UGGAAGACUAGUGAUUUUGUUGU----
dme-miR-7     --UGGAAGACUAGUGAUUUUGUUGU----
dpu-miR-7     --UGGAAGACUAGUGAUUUUGUUGU----
cqu-miR-7     --UGGAAGACUAGUGAUUUUGUUGU----
csa-miR-7     --UGGAAGACUAGUGAUUUUGUUGU----
bfl-miR-7     --UGGAAGACUAGUGAUUUUGUUGU----
bmo-miR-7     --UGGAAGACUAGUGAUUUUGUUGU----
ame-miR-7     --UGGAAGACUAGUGAUUUUGUUGU----
aga-miR-7     --UGGAAGACUAGUGAUUUUGUUGU----
aae-miR-7     --UGGAAGACUAGUGAUUUUGUUGU----
ssc-miR-7     --UGGAAGACUAGUGAUUUUGUUGUU---
lgi-miR-7     --UGGAAGACUAGUGAUUUUGUUGUU---
bta-miR-7     --UGGAAGACUAGUGAUUUUGUUGUU---
cte-miR-7     --UGGAAGACUAGUGAUUUUAUUGUU---
bma-miR-7     --UGGAAGACUUGUGAUUUUGUUGU----
cis-miR-7a    --UGGAAGACUGGUGAUAU-GUUGUUUUU
cis-miR-7c    UAUGGAAGACUGGUGAUAU-GUUGUU---
cis-miR-7b    --UGGAAGACUGGUGAUAU-GUUGUUAGA
                ******** ***** *   **
```

```
aae-miR-133     -UUGGUCCCCUUCAACCAGCUGU-
bfl-miR-133     --UGGUCCCCUUCAACCAGCUGUA
aga-miR-133     -UUGGUCCCCUUCAACCAGCUGU-
ame-miR-133     -UUGGUCCCCUUCAACCAGCUGU-
bmo-miR-133     -UUGGUCCCCUUCAACCAGCUGU-
cte-miR-133     -UUGGUCCCCUUCAACCAGCUGU-
cqu-miR-133     -UUGGUCCCCUUCAACCAGCUGU-
lgi-miR-133     -UUGGUCCCCUUCAACCAGCUGU-
nvi-miR-133     -UUGGUCCCCUUCAACCAGCUGU-
sko-miR-133     -UUGGUCCCCUUCAACCAGCUGU-
dme-miR-133     -UUGGUCCCCUUCAACCAGCUGU-
tca-miR-133     -UUGGUCCCCUUCAACCAGCUGU-
dpu-miR-133     -UUGGUCCCCUUCAACCAGCUGU-
isc-miR-133     -UUGGUCCCCUUCAACCAGCUGU-
sja-miR-133     -UUGGUCCCUAUCAACCAGCUGU-
cin-miR-133     -UUGGUCCCCUUCAACCAGCUG--
spu-miR-133     UUUGGUCCCCUUCAACCAGCCGU-
cis-miR-133a    UUUGGUCCCUUUCAACCAGCUA--
cis-miR-133b    UUUGGUCCCUUUCAACCAGCUA--
bma-miR-133     -UUGGUCCCCUUCAACCAGCUA--
tgu-miR-133     UUUGGUCCCCUUCAACCAGCU---
fru-miR-133     UUUGGUCCCCUUCAACCAGCUG--
tni-miR-133     UUUGGUCCCCUUCAACCAGCUG--
                  *******  *********
```
